# T Cell Memory in Infection, Cancer, and Autoimmunity

**DOI:** 10.3389/fimmu.2021.811968

**Published:** 2022-01-03

**Authors:** Vincenzo Barnaba

**Affiliations:** ^1^ Istituto Pasteur Italia, Fondazione Cenci Bolognetti, Rome, Italy; ^2^ Dipartimento di Scienze Cliniche, Interistiche, Anestesiologiche e Cardiovascolari, Sapienza Università di Roma, Rome, Italy

**Keywords:** adaptive immunity, immunological memory, infection, vaccination, cancer, autoimmunity

## Abstract

Long-term immunological memory represents a unique performance of the adaptive immunity selected during evolution to support long-term survival of species in vertebrates, through protection against dangerous “invaders”, namely, infectious agents or unwanted (e.g., tumor) cells. The balance between the development of T cell memory and various mechanisms of immunoregulation (namely, T cell effector exhaustion and regulatory T cell suppression) dictates the fate in providing protection or not in different conditions, such as (acute or chronic) infection, vaccination, cancer, and autoimmunity. Here, these different environments are taken in consideration to outline the up-to-date cellular and molecular features regulating the development or damping of immunological memory and to delineate therapeutic strategies capable to improve or control it, in order to address pathological contexts, such as infection, tumor, and autoimmunity.

## Introduction

Unique and essential properties of the adaptive immune system are the fine specificity towards each type of peptides (epitopes) and the long-term immunological memory. The latter usually develops following resolution of a given infection, through the generation of memory B and T cells, which persist for almost a lifetime and promptly trigger secondary protective responses in the event of reinfection (1–3). The adaptive immune system and the concatenated long-term immunological memory (appearing about 450 million years ago in fish) were selected in the vertebrates in a Darwinian fashion, probably under the evolutive pressure of the significantly higher lifespan and lower reproductive capacity, as compared with the invertebrates only having the innate immunity (present since 1 billion years ago without having had any fundamental changes to date): for instance, insects do not need the long-term memory of an infection, because they have a very short life and a huge number of offspring allowing the species survival. The first description of the immunological memory was probably dated back to Thucydides, who reported, in his writing about the plague outbreak that decimated the Athenians during the Peloponnesian war against Sparta (443–404 BC), that individuals who recovered from the infection no longer get sick.

**Figure 1:**
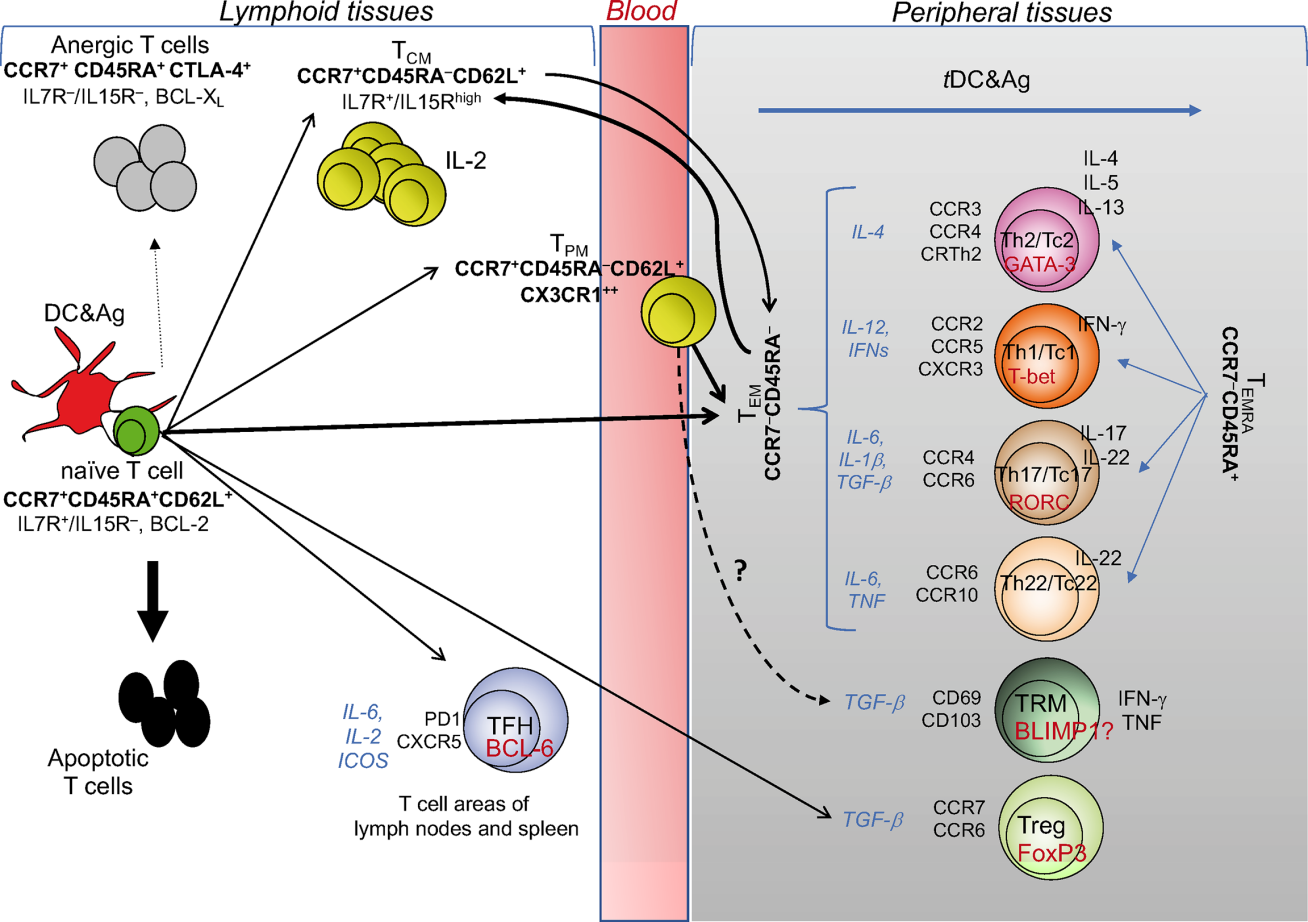
T cell diversity and memory pathways. This cartoon depicts the “one cell, multiple fates” hypothesis proposing that a single naïve (CD4^+^ or CD8^+^) T cell undergoes multiple fates, according to the strength of signals 1 and 2 received by pAPCs: suboptimal signals (e.g., signal 1 without signal 2) causes T cell anergy; excessive signals (as in the case of response to superantigens, such as some bacterial toxins) cause T cell apoptosis; optimal signal strength induces typically and functionally distinct T_EM_, T_CM_, and T_PM_ cells with the same specificity. T_CM_ cells can also derive from T_EM_: upon performing effector functions, the majority of T_EM_ dye, while a minority is saved and generates T_CM_. T_CM_ cells acquiring the chemokine lymphoid homing receptors (indicated in bold under the T_CM_ subset) continuously recirculate *via* the bloodstream to lymphoid organs, and promptly generate secondary responses. CX3CR1^+^ T_PM_ cells migrate from blood to tissue to lymph nodes, proliferate in a higher fashion than the T_CM_ population, display the ability to perform cytotoxic function, and survey non-lymphoid tissues. The differentiation into the various T_EM_ subsets (Th1, Th2, Th17, Th22, or the cytotoxic T [Tc] cell counterparts) is conditioned by the microenvironmental cytokine milieu (indicated by the cytokines in italics), in which the response takes place. Under these conditions, each of the T_EM_ subsets activates its own specific transcription master regulator (indicated in red within each T_EM_ subset) contributing to establish the gene expression patterns correlated with specific cytokine immune-phenotypes (indicated to the right of each T_EM_ subset), and expresses its own specific pattern of inflamed tissue homing receptors (indicated to the left of each T_EM_ subset). Upon migration into the specific inflamed tissues, each type of T_EM_ subset is ready to differentiate into terminally-differentiated effector cells (T_EMRA_) promptly performing the functions for which they are programmed (Th2, Th1, Th17, etc.), following contact with tissue-resident (*t*)DCs presenting the specific antigens derived from various type of pathogens: that is, the helminths preferentially will condition the Th2, the intracellular pathogens the Th1, the fungi the Th17 differentiation, etc. TFH cell differentiation is regulated by IL-6, IL-2, inducible costimulator receptor (ICOS): if the chemokine receptor CXCR5 is expressed, they will migrate to the border of the B cell follicle and help B cell differentiation, whereas, in the presence of the related cell signals, they differentiate into Th1-, Th2-, or Th17-like cells, exit the lymphoid tissue and traffic to the site of infection or inflammation. Similar cell diversification occurs upon optimal activation of CD8^+^ T cells that also acquire the cytotoxicity function (Tc: T cytotoxic cell). T_RM_ and Treg profiles are conditioned by the antigens they meet directly in the tissues and lymphoid organs, respectively, in the presence of TGF-β.

The generation of effective adaptive immune responses against the “invaders”, which come from outside or are aberrantly generated in our body (principally infecting agents or tumors), requires that naïve B or T lymphocytes receive the appropriate signals (i.e., antigenic signal 1 and costimulatory signal[s] 2) provided by professional (p) antigen-presenting cells (APCs) (1–3). In this review, we will focus on T cell responses. pAPCs (principally the myeloid or monocyte-derived dendritic cell [DC] subsets normally patrolling our tissues), mature and convert from tolerogenic into stimulatory (s)DCs, following the exposure to the innate immunity (infectious or danger) signals (signal[s] 3) received within inflamed tissues. sDCs more efficiently phagocytose and process antigens, upregulate both major histocompatibility complex (MHC) and costimulatory molecules, and acquire the capacity to efficiently migrate into draining lymphoid organs, because of the overexpression of appropriate homing chemokine receptors (e.g., CCR7). Once arriving into lymph nodes, sDCs efficiently present or cross-present peptides (generated by the antigen processing) on class II or class I MHC molecules to high avidity T cell receptors (TCRs) of CD4^+^ or CD8^+^ T cells, respectively (signal 1), and provide various costimulatory molecules (e.g., B7.1, B7.2, B7RP1, CD27 ligand [L]) interacting with the corresponding receptors (e.g., CD28, ICOS, CD27) expressed by naïve T cells (signal 2) (**Figure 1**). Only under these conditions, the single antigen-specific naïve T cells, whose frequency ranges between about 1/200,000 and 1/1,000,000 cells in humans according to the type of antigen (4), are primed, proliferate by several logs of magnitude, differentiate into protective effector cells clearing the “invader”, and generate long-term memory. Basically, CD4^+^ T helper (h) cells and CD8^+^ T cytotoxic (c) cells divide the labor: the former help B cells to produce long-lived antibody responses, synthesize a wide variety of cytokines (depending on the context) and, in some setting, can acquire cytotoxic function; the latter are primarily antigen-specific cytotoxic cells, and can produce various types of cytokines, such as the CD4. The requirement of third party CD4^+^ T cells in the interaction DCs/CD8^+^ T cells for CD8^+^ T cell priming and long-term CD8^+^ T cell memory development (5–7) is inversely correlated with the level of inflammation and pathogen- or danger-associated molecular pattern (PAMP or DAMP) signals conditioning the priming (3, 8). It is superfluous in high-level inflammatory contexts, in which PAMP or DAMP signals trigger a variety of inflammatory responses and mediate DC activation resulting in CD8^+^ T cell priming and memory development (9–13). By contrast, the need of CD4^+^ T cell help for priming CD8^+^ T cell responses seems to be necessary in chronic infections, where various signals (2 and/or 3) are impaired (3), although it still fails to restore effective CD4^+^ or CD8^+^ T cell memory.

The generation of long-term immunological memory is dependent on appropriate level of immunopathology caused by the innate and adaptive effector immune responses addressed to eliminate the “invader” by killing infected host cells and providing tissue inflammation that stop upon pathogen clearance (recovery). The great value of vaccination is based on its capacity, through the administration of “invader” antigens (signal 1) and adjuvants (such as alum, MF59, ASOs, CpG, TLR ligands, viral, RNA or DNA vectors) (14) (signal 3 principally addressed to activate DCs providing signal 2), to elicit strong immune responses and long-term memory mimicking those observed in individuals recovered from a natural infection, without the severe phenomena associated with the disease that can even result in death.

## T Cell Diversity

The first seminal report on the diversification of memory T cells was by Sallusto and Lanzavecchia (15), showing that memory T cells can be subdivided by distinct expression pattern of adhesion molecules and chemokine receptors allowing different migratory pathways. Naïve T (T_N_) cells and central memory T (T_CM_) cells (both expressing high-level of lymphoid homing markers CD62L and CCR7) continuously recirculate *via* the bloodstream to lymphoid organs. In addition, T_CM_ cells persist by an IL-7 and/or IL15-dependent homeostatic proliferation, without the antigen persistence, produce high IL-2 levels and display high self-renewal/proliferation potential upon antigen re-encounter (16–18). By contrast, the various functional subsets of effector memory T (T_EM_) cells lose the high proliferation potential and the lymphoid homing markers, acquire diverse patterns of inflamed tissue homing markers (see classification of T cell subsets in **Figure 1**) and display prompt effector functions, according to the type of peripheral tissue and cytokine milieu, in which they differentiate and migrate (**Figure 1**) (19). Recently, the surface expression of the chemokine receptor CX3CR1 has been used to better classify effector and memory T cells (20, 21). CX3CR1 identifies a subset termed peripheral memory T cells (T_PM_) that migrate from blood to tissue to lymph nodes, show higher self-renewal capacity and proliferation than the conventional T_CM_ population, display the ability to perform cytotoxic function, and survey non-lymphoid tissues. In parallel, a subset of effector CX3CR1^high^ T cells appears primarily restricted to the intravascular space and spleen and represent a major source of T_EM_ cells. Furthermore, a pool of tissue-resident memory CD8^+^ T (T_RM_) cells has been more recently identified (22). T_RM_ cells persist long term in non-lymphoid tissues, express a transcriptional signature shared with both T_CM_ and T_ME_ cells that can be conditioned by individual tissues in which they survive, and control possible foreign “invasions” by recruiting other immune cells and triggering inflammatory processes.

Recent technological advances tracing CD8^+^ T cells at single-cell level in mouse *in vivo* support the “one cell, multiple fates” hypothesis, according to which a single naïve T cell (with a single specificity) generates multiple phenotypically and functionally distinct effector and memory T cells with the same TCR (23) (**Figure 1**). However, we cannot completely exclude the “one cell, one fate” hypothesis, dictating that single T cell clones with various degrees of affinity for a given peptide select unique fates for each single clone: for instance, a clone will become Th1, another Th2, still another Th17, etc. (23). Consistently, by combining antigenic stimulation and TCR deep sequencing technologies, it has been elegantly proposed that CD4^+^ T cell responses can develop according to both the hypotheses in humans. Indeed, single naïve or memory CD4^+^ T cells primed by various pathogens (*Candida albicans*, *Mycobacterium tuberculosis*, *tetanus toxoid*) *in vitro* can undergo multiple fates, that is Th1, Th2, and Th17 cells with different migratory capacity, comprising both clones polarizing toward a single fate, and clones whose progeny acquire multiple fates (24) (**Figure 1**). The stochastic combination of several events (e.g., TCR affinity and costimulatory signals, the cytokine milieu, the type and dose of antigens, the duration of antigen exposure) may condition the different (single or multiple) fates in the context of the same polyclonal immune responses. Altogether, these multiple epigenetics-driven fates provide a high level of plasticity to the single memory T cells, which can thus employ different and prompt alternative strategies to fight and eliminate each type of pathogens and to maintain long-term memory.

Because the generation of the different memory T cell subsets after infection (or in response to vaccination) is principally addressed both to eliminate the “invader” (recovery) and to recall rapid secondary responses in the case of reinfection (memory), a main question at the center of immunology research is: how is the T cell diversification regulated and capable to provide immunological memory in condition of chronic (long-lasting) self- or non-self-antigen stimulation, as it happens in the course of chronic infections, tumors, and autoimmune diseases?

Addressing these questions is of pivotal importance to understand the basic role of adaptive T cell immunity and memory and their implications in infection resolution, effective vaccination, chronic infection, cancer, or autoimmunity, in order to develop new therapeutic strategies (tuning of immune responses by biologicals, adaptive immunotherapy, vaccination) in the different clinical conditions.

## T Cell Diversity in Acute Infections or Vaccination

**Figure 2:**
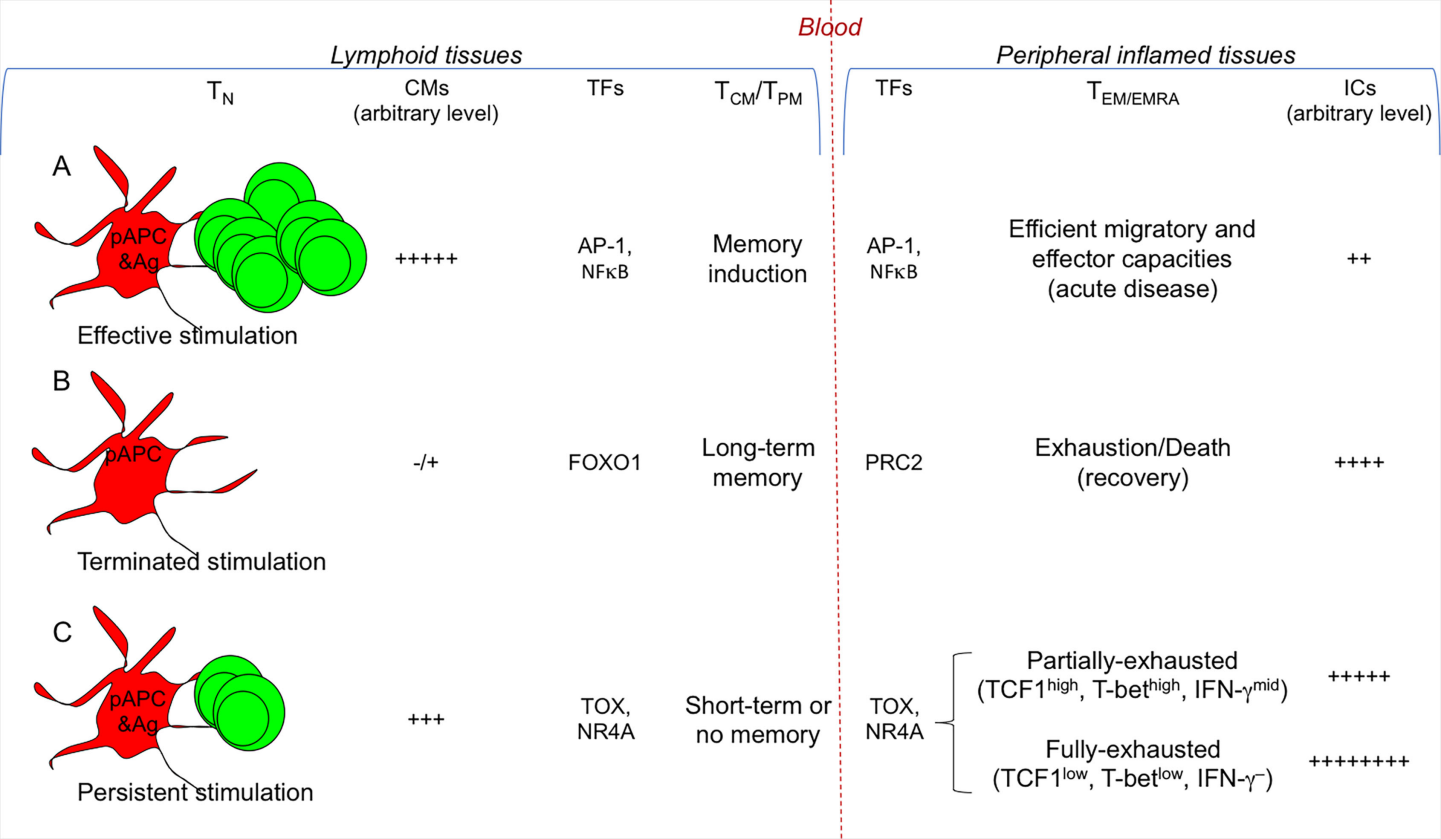
Signals conditioning T cell diversification. Different strengths of T cell stimulation result in different fates. The most relevant TFs involved are indicated. **(A)** Efficient and transient antigenic and co-stimulatory molecule (CM) signals by pAPCs condition strong T cell proliferation and differentiation into memory T cells (T_CM_/T_PM_) and the various types of T_EM/EMRA_ cell subsets (Th/c1, Th/c2, Th/c17,…), selected according to the cytokine milieu in which the response takes place (see **Figure 1** ). TFs (e.g., AP-1, NFκB) activate memory (e.g., *IL7R*, *BCL2*) and effector genes (e.g., *IFNG*, *GZMB*). **(B)** Under these conditions, the T cell response generally results with the eradication of a given pathogen and recovery: the combination of termination of antigen exposure and upregulation of ICs on T_EM/EMRA_ cells will lead to their drastic decrease and death, whereas memory cells will persist without the presence of antigen, and will provide long-term memory. TFs such as FOXO1 maintain long-term memory, whereas PRC2 contributes in silencing effector genes. **(C)** In the presence of persistent antigen-stimulation (e.g., chronic infections or tumors), the continuous viral or tumor mutations will induce the generation of continuous waves of T cells, which, because chronically exposed to antigen-stimulation, will upregulate a wide repertoire of ICs, and will undergo chronic T cell exhaustion resulting in the lack of (long-term) immunological memory. TFs (e.g., TOX, NR4A) favor the transcription of exhaustion genes (e.g., encoding PD-1, TIM-3, CTLA-4), overwhelming the work of those (e.g., AP-1, NFκB) activating effector genes (e.g., *IFNG*, *GZMB*) or memory genes (e.g., *IL7R*, *BCL2*). In the early phase of the persistent antigen-stimulation, T_EM/EMRA_ cells will become partially-exhausted (TCF1^high^, T-bet^hgh^, IFN-γ^mid^), then they will degenerate in fully-exhausted cells (TCF1^low^, T-bet^low^, IFN-γ^–^) and will further upregulate ICs. Partially-exhausted T cells can be rescued by ICB.

The resolution of most acute infections or the vaccination against the related pathogens (e.g., smallpox, mumps, rubella, chickenpox, measles, diphtheria, polio, meningococci, hepatitis A virus [HAV], HBV) correlate with protective adaptive effector responses (i.e., neutralizing antibodies and effector CD4^+^ and CD8^+^ T cells) and development of long-term memory (**Figures 2A, B**). In particular, high affinity TCRs and the coreceptors (CD4 or CD8) on naïve T cells, following receiving sustained antigenic signals 1 by pAPCs, deliver the signaling cascades through the phosphorylation of multiple consecutive molecules (e.g., ITAM, ZAP70, LCK, LAT, PLCγ, IP3,…), ultimately leading to the nucleus translocation of various transcription factors (TFs) (e.g., NFκB, NAFT family) that, through their own conserved DNA binding domains, favor the expression of a wide series of genes associated to T cell activation and memory (25–28). The costimulatory molecules on naïve T cells (engaged by the respective ligands expressed on APCs [signal 2]) amplify the activation signal cascade, through the phosphorylation of additional messengers (e.g., PIK3, ERK, RAS…) essential for T cell priming, without which signal 1 alone could cause T cell anergy. The effective TCR signal strength must be transient and not persistent, to avoid a prolonged expression of genes associated with the “T cell exhaustion” (e.g., *Pdcd1* or *CTLA4* encoding the inhibitory receptors PD-1 and CTLA-4, respectively), and to guarantee thus the full T cell effector responses and the generation of long-term memory. The short duration of the TCR signaling has been proposed to induce a transient DNA demethylation of the *Pdcd1* locus (encoding PD-1), followed by *Pdcd1* re-methylation that coincides with efficient effector functions addressed to fight the “invaders” (29). In the late phase of activation (i.e., in the absence of antigen-stimulation due to the pathogen clearance), T cells upregulate a wide repertoire of inhibitory signals (i.e., immune check-points [ICs], such as CTLA-4, PD-1, TIM-3, LAG-3, TIGIT, VISTA), which, following contact with the respective ligands expressed by both lymphoid and non-lymphoid cells, deliver an inhibitory cascade leading to the dephosphorylation of the molecules associated with the TCR, co-receptor, and co-stimulatory signaling (30). This intrinsic immunoregulatory mechanism was likely selected during evolution to terminate the immune responses that would be useless if not harmful, when a given infection cleared. Under these conditions (that is, the combination of termination of antigen exposure and IC expression), effector T cells drastically decrease and die upon performing their (protective) effector functions, whereas the sister memory cells selected by specificity and function persist without the presence of antigen, and promptly respond on demand, by generating new waves of effector immune responses in the case of reinfection (**Figure 2B**). Therefore, the stop signals contributing to the “crash” of effector responses, can also contribute to develop immunological memory (**Figures 2A, B**).

The generation and distribution of each of these memory T cell subsets obey highly diverse epigenetic, transcriptional and proteome pathways (2, 19, 23, 31–33). The studies on genome-wide transcriptional and epigenetic changes (by using the assay for transposase-accessible chromatin with sequencing (34) during infection or vaccination showed that DNA methylation, histone modifications, and transcriptional signatures diversifies T cell effector and memory differentiation. These analyses in mouse models revealed that long-lived memory T cells have a naïve-like transcriptome but an effector-like open chromatin map (i.e., demethylation of *IFNG* and *GZMB* genes and an open chromatin near their promoters), suggesting a mechanism by which memory T cells are equipped to rapidly perform effector functions (35). *Vice versa*, open chromatin regions were found in the *IL7R* and *BCL2* genes in both naïve and memory cells, but not in effector cells, suggesting that memory cells conserve important molecular features of naïve cells, associated to survival and self-renewal (**Figure 2A**). In addition, various studies demonstrated that active transcriptional maintenance by FOXO1 is required to sustain memory T cell longevity and self-renewal, whereas the epigenetic factor PRC2 contributes in silencing genes associated with terminally-effector T cells, following infection resolution (36–39) (**Figure 2B**).

An unresolved question regards the generation of long- or short-term memory, after different types of infection or vaccination. The majority of the current vaccines are administrated subcutaneously and cause long-term memory against the pathogens towards which they are directed (e.g., smallpox, mumps, rubella, chickenpox, measles, diphtheria, polio, meningococci, HAV, HBV). By contrast, the immunological memory resulting upon SARS-CoV-2, various common cold or influenza virus infections or the related vaccinations is generally short-term, likely because a much higher production of specific immune responses at the level of the upper respiratory tracts of the lung would be needed to generate long-lasting protection in these infections (40). In particular, vaccines administrated through the mucosal airways (the gateway to viruses such as SARS-CoVs, common colds or influenza) should likely generate more efficient immune responses in these sites than the current vaccines administrated subcutaneously, and favor long-term memory. These hypotheses could be confirmed by using single-cell sequencing technology, allowing to analyze the complexed module, namely, the transcriptional pathways, the level of transcription factors, and the chromatin accessibility in various immune cell types after the different infection recoveries or vaccination types.

## T Cell Diversity in Chronic Infection and Cancer

The sustained antigenic stimulation provided by persisting infection or cancer breaks the fine molecular balance conditioning the protective effector responses and the generation of long-term memory by various interacting mechanisms, namely, viral or tumor immune escape, T cell exhaustion, and suppression by regulatory T cells (Tregs).

### Immune Escape and T Cell Diversity in Chronic Infection and Cancer

A principal mechanism of immune escape evading both T and B cell recognition and affecting T cell diversity is caused by viral or tumoral mutations, resulting in the establishment of a state of chronic low-level immunopathology that, despite unable to clear the persisting virus or tumor, delays ultimately the “catastrophe” (i.e., failure of chronically-infected organs or rapid spread of metastatic tumors) as much as possible. The mechanisms establishing chronic low-level immunopathology are likely selected, during the evolutionary process, to allow a long-term survival of the host (i.e., compromise between the host and the persisting viruses or tumors), by avoiding excessive damage of normal tissues, on the one hand, and excessive virus or tumor spread in the body, on the other hand (41).

Persisting viruses, such as HCV (a single strand RNA virus causing chronic hepatitis in 60–80% of infected individuals, depending on the geographical areas), HIV-1 (a lentivirus belonging to the *Retroviridae* family, infecting human immune cells and causing AIDS in the majority of infected individuals without treatment), and to a lesser extent HBV (a double strand DNA virus causing chronic hepatitis in less than 3% of infected individuals), provide different mutation rates that can have equally different impact on BCR or TCR diversity. The lack of proofreading activity by HIV-1 reverse transcriptase or by HCV-RNA-dependent RNA polymerase makes replication of HIV-1 or HCV (in contrast to HBV) extremely error-prone: these errors have been estimated in a range of 1 mutation in 1,000 to 100,000 nucleotides per replication cycle for RNA viruses (e.g., HCV or HIV-1), and approximately 1 mutation in 100,000,000 nucleotides per replication cycle for DNA viruses (e.g., HBV) (42, 43). These differences in the mutational fitness can contribute to the capacity of HCV or HIV-1 to escape from the huge B or T cell repertoire specific to the “wild-type” viral epitopes and to establish chronic infection much more frequently than HBV, and, as a consequence, to the efficient development of immunological memory in the latter, as compared with the former. By contrast, the capacity of the coronaviruses, including SARS-CoV-2, to proofread and clear mismatched nucleotides during replication (44), leads to hypothesize that these viruses cannot persist and establish chronic infection because of the low mutation rate, although the evident epidemiological role of these mutations. The lack of long-term immunological memory in these infections is likely due to the rapid subversion of mucosal immunity (innate and then adaptive) at the level of the gate entry (i.e., upper respiratory tracts).

As well as persisting viruses, “hot” tumors, such as melanoma or non-small cell lung cancer (NSCLC) that, in contrast to “cold” tumors, are characterized by significant DNA instability, principally due to the lack of mismatching repair mechanisms, show a very high mutational burden generating a huge repertoire of mutated (passenger) neoantigens, and a high number of tumor-infiltrating T cells (TILs) (45, 46). T cells specific to these mutated neoantigens, which are not purged by central tolerance, can migrate in the periphery, massively infiltrate hot tumors and be of particular relevance to tumor control (47, 48). Therefore, regardless of the origin of mutated (viral or tumor) antigens, the immune system is equipped to chase the continuous viral or tumor mutations through the generation of equally continuous new waves of mutated antigen-specific T cell clones (49). However, the generation of the huge repertoire of mutations can escape from B and T cells and contribute to the tumor mutational fitness and to the difficulty in developing effective immunological memory.

### T Cell Exhaustion and Memory in Chronic Infection and Cancer

In the course of chronic infections or tumors, T cells will be unable to eliminate the persisting (hyper-mutational) virus or tumor, upregulate a wide repertoire of ICs, and, in the long run, will undergo the combination of a T cell dysfunctional state defined “T cell exhaustion”, and the lack of long-term memory, resulting ultimately in irreversible chronic infection or tumor progression (41) (**Figure 2C**).

The molecular bases of T cell exhaustion and absence of long-term memory include a multitude of simultaneous and progressive transcriptional and epigenetic events. First, the long duration of TCR signaling by persistent antigens has been demonstrated to lead to a complete demethylation of the *Pdcd1* regulatory region that remains persistently unmethylated, and impedes thus the re-stabilization of efficient effector functions, as in the case of short duration of TCR signaling, shown in resolving infections (29, 50). Then, various types of histone modifications lead to a state, in which the chromatin is stably open and accessible to a multitude of TFs (e.g., TOX, NR4A) favoring the transcription of exhaustion genes (e.g., encoding PD-1, TIM-3, CTLA-4), and overwhelming the work of those (e.g., AP-1, NFκB,…) activating effector (e.g., *IFNG*, *GZMB*) or memory (e.g., *IL7R*, *BCL2*) genes (35, 51, 52) (**Figure 2C**).

Depending on the time these processes start, they may or may not be restored. In the early phases of persistent stimulation, exhausted T cells (PD-1^+^CTLA-4^+^TIM-3^+^…) can be rescued principally if they express a further TF, the TCF1 encoded by the *TCF7* gene: TCF1^high^ cells express the master TF for IFN-γ production T-bet, at a level enough for producing moderated levels of IFN-γ (partially-exhausted T cells), although not at the levels observed in resolving infections, and may hence contribute to maintain the state of chronic low-level inflammation (53–57). The TCF1^high^ T cells can be efficiently rescued and acquire a stronger effector profile and anti-tumor activity by the treatment with IC inhibitors (e.g., anti-PD-1, anti-PDL-1, anti-CTLA-4 mAbs), or the combination of the latter with vaccine therapy containing mutated tumor neo-antigens (58). By contrast, in the late exhaustion phase, T cells become TCF1^low^, acquire a fully-exhaustion phenotype (PD-1^high^CTLA-4^high^TIM-3^high^), and, as a consequence, cannot be rescued by IC blockade (ICB) or vaccination therapies, likely because the TFs favoring expression of ICs have stably blocked the chromatin accessibility to the TFs favoring effector and memory gene expression (53). Therefore, ICB (better if associated with possible therapeutic vaccines) can provide extraordinary beneficial effects in early hot tumors rather than in very late tumors or chronic infections, where the majority of TILs will have become fully-exhausted.

A consistent proportion of tumor neoantigens can also be non-mutated neoantigens, when they derive from various forms of protein modifications occurring at post-transcriptional level in tumor cells, such as protein splicing, dysregulated phosphorylation or glycosylation, proteasome generation of spliced peptides, peptide citrullination, impaired peptide processing in TAP-deficient tumor cells, or proteasomal degradation of defective ribosomal products (59–65). These non-mutated neoantigens may provide rational targets for cancer immunotherapy, because they should not be expressed or expressed at concentrations that are not enough to delete specific T cells in the thymus. In addition, also chemotherapy- or radiotherapy-based apoptosis of tumor cells, and also providing various danger signals (e.g., ATP, UTP, calreticulin, HMGB1) that activate DCs and can strengthen T cell priming and memory (immunogenic cell death) (66), enable tumor cells to unveil non-mutated neoantigens, in the form of caspase-cleaved antigenic fragments (67). A wide variety of them has been recently identified by using stable isotope labeling by amino acids in cell culture-based mass spectrometry in human NSCLC cells, namely, caspase-cleaved fragments from olfactory receptor 5H2, Ras and EF-hand domain-containing protein, proactivator polypeptide, protein LYRIC, zinc transporter SLC39A7, ADP/ATP translocase 2, chatepsin D, and ruvB-like 2 (67). These caspase-cleaved fragments were upregulated only in apoptotic tumor cells, targeted to the processing machinery and cross-presented in form of peptides by APCs much more efficiently than their entire protein counterparts, supporting their definition of tumor non-mutated neoantigens (67). The immunogenicity of these non-mutated neoantigens is proved by the evidence that CD8^+^ T cells specific to the related epitopes were significantly represented in NSCLC patients following chemotherapy treatment, increased in their frequency upon ICB therapy, and correlated with overall survival, suggesting their contribution in the tumor control and possibly in the immunological memory improvement (67).

### Tregs and T Cell Memory in Chronic Infection and Cancer

Under conditions of long-lasting tumors or chronic infections, which are characterized by impaired effector and memory responses principally due to the irreversible T cell exhaustion, other immunosuppressive mechanisms amplified and are intertwined. First of all, the intervention of various subsets of Tregs, namely, CD4^+^ Tregs expressing the master transcription factor FOXP3 (68, 69), can be either committed in the thymus (thymus-derived Tregs) or induced in the periphery [as reviewed in (70)], or the suppressor CD8^+^ T cell subset representing, historically, the most ancient population with suppression function described (71–74). Regardless of the cell lineage, Tregs can provide various homeostatic effects that can result in being beneficial or detrimental, depending on the setting in which they govern the homeostasis. Tissue-resident CD4^+^ or CD8^+^ Tregs perform tissue-protective activities, by promoting tissue repair, systemic metabolism, and immunosuppression, particularly by the production of TGF-β or IL-10 (75, 76). These activities are beneficial in resolving acute inflammatory diseases by promoting tissue health, but become detrimental in chronic inflammatory diseases, because they contribute to organ failure *via* the persisting tissue repair mechanisms, resulting in tissue subversion (e.g., fibrosis and cirrhosis) and tumor development. In addition, the beneficial effects by Tregs, for which they have evolutionarily selected, are based on their primary function to prevent the differentiation of autoreactive T_N_ cells into harmful effector cells (avoiding thus autoimmunity) in the periphery (peripheral tolerance), and to stop or limit the excessive immunopathology by self- or non-self-reactive T effector cells through a wide range of immunosuppressive mechanisms (77, 78). Again, these immunoregulatory effects can result in being detrimental in the course of chronic infections or tumors, because FOXP3^+^ Tregs acquire strong suppression capacity in these contexts, through various signals (e.g., by interaction between OX40L expressed on tumor-associated macrophages and OX40 delivering survival signals in Tregs) favoring demethylation of the Treg-specific demethylated region that acts as a transcriptional stabilizer of *FOXP3* gene and consequent suppression function [as reviewed in (70, 79)]. In addition, Tregs in stable tumors or chronic infections receive signals from the tumor microenvironment that provide supplemental energetic routes involving lipid metabolism, conferring a preferential proliferative advantage to Tregs (80). Interestingly, the excessive Treg improvement can be limited by Treg intrinsic mechanisms that try to govern the excess suppression, in order to contribute to slow down the progression of chronic infections or tumors. The first report describing counter-suppression of FOXP3^+^ Tregs showed that the interaction between PD-1 and PD-L1, both expressed on well-stabilized activated FOXP3^+^ Tregs, provided a negative signal into these Tregs by PD-1 limiting STAT-5 phosphorylation and Treg expansion and suppression (81). Conversely, other studies demonstrated that PD-1 and also TIM-3, contribute to the conversion of naïve CD4^+^ T cells into induced FOXP3^+^ Tregs through various molecular pathways, namely, the capacity of PD-1 signaling to inhibit the sparaginyl endopeptidase enzyme normally cleaving FOXP3 in induced Tregs [reviewed in (82)]. Therefore, PD-1 may act as a double-edged sword with the effect dependent on the phase of Treg activation: it contributes to induce Tregs from conventional naïve CD4^+^ T cells, on the one hand, and to downregulate stable Treg expansion and functions, on the other. The counter-suppression effect by PD-1 on stable activated Tregs could in turn be countered by ICB treatment, improving Treg proliferation and suppression (81). This data suggests to use ICB carefully both to avoid the detrimental effects by rescued Tregs resulting in “hyperprogression” of tumors (or chronic infections) by excessive suppression of protective effector T cells [reviewed in (83)], and to employ ICB selectively in tumors (and likely chronic infections) expressing PD-1 on CD8^+^ TILs rather than on Tregs (84).

Collectively, the various mechanisms that cause effective Treg-mediated suppression contribute to get worse the T cell dysfunctional state and to impede the long-term memory development, resulting ultimately in irreversible chronic infections or tumor progression.

### May T Cell Memory be Restored in Chronic Infection or Cancer?

An open question is: can exhausted T cells upon elimination of chronic antigenic stimulation or that have been restored by ICB in terms of effector functions, differentiate into long-term memory cells? Chronically-infected HCV patients following virological cure by direct antiviral agents allowing complete HCV clearance, and also mouse models of chronic viral infection, showed that upon eliminating the virus, TCF-1^+^ exhausted T cells downregulate ICs and partially acquire phenotypic and transcriptional features of memory-like cells (85–87). Importantly, T cells that were exposed to HCV antigens for less time were functionally and transcriptionally more similar to memory T cells from spontaneously resolved HCV infection (87, 88). These data confirm that exhausted T cells may or not may be (at least partially) restored providing differentiation of memory-like cells, depending on the time in which the process initiated and on the frequency of TCF1^+^ exhausted T cells (53–57). However, functionally, exhausted T cells, which have been rescued by the elimination of chronic antigenic stimulation, maintain critical transcriptional regulators in the exhaustion state, and their recall capacity remained limited and not durable over time as compared to true memory T cells from competent mice (85). Chromatin-accessibility profiling revealed a failure to recover memory epigenetic circuits and maintenance of a largely exhausted open chromatin landscape, constraining the establishment of long-term immunological memory (85). We cannot exclude that a longer time of antigen-free recovery can reinvigorate previously exhausted CD8^+^ T cells that can persist and respond to reinfection (89). More in depth molecular analyses, particularly at epigenetic level, need to discriminate these possibilities in order to delineate new therapeutic interventions addressed to develop immunological memory in chronic infections and tumors.

**Figure 3:**
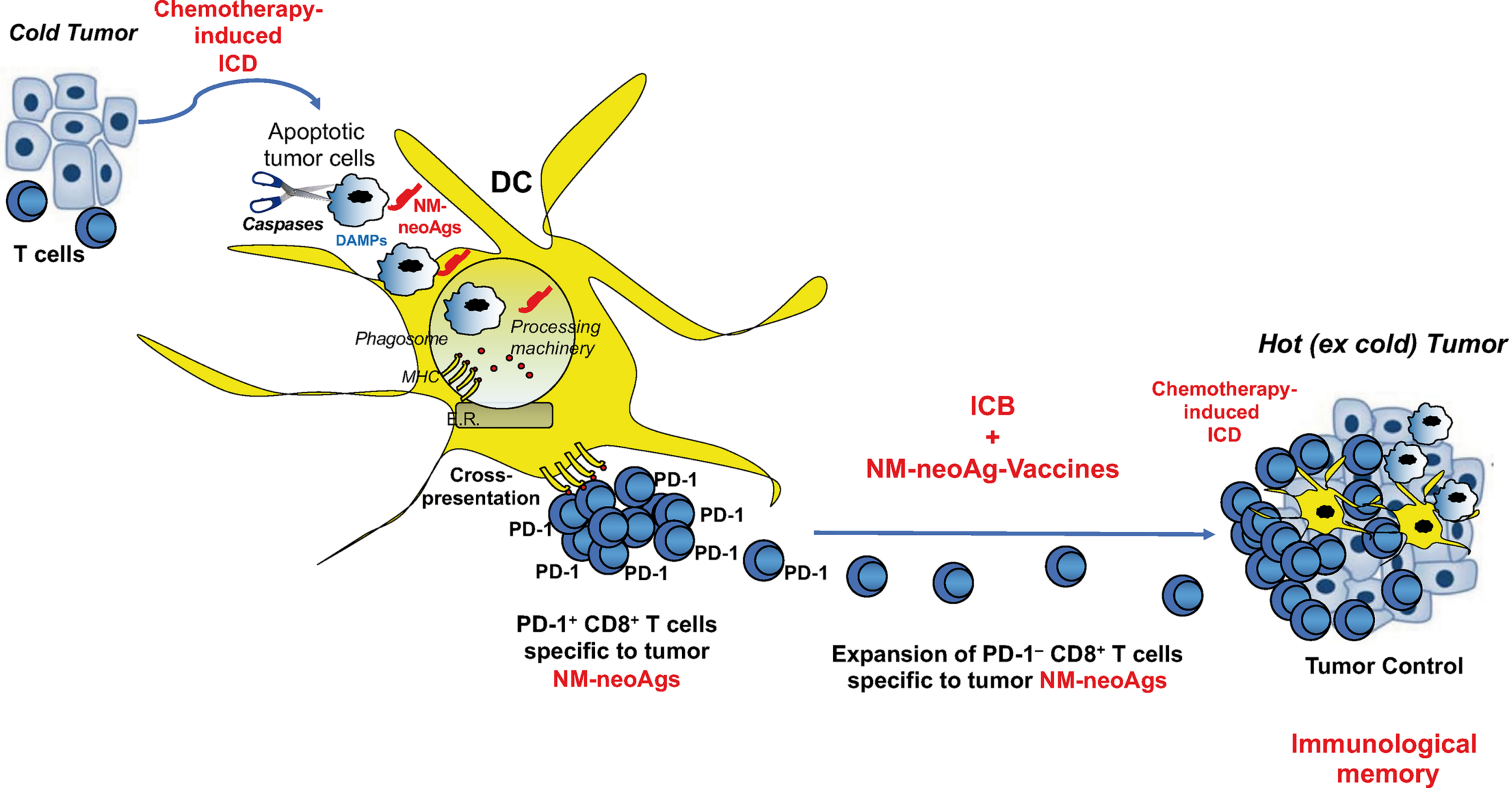
Combination of immunogenic cell death, non-mutated neoantigen-based vaccination, and ICB in tumor immunotherapy. Immunogenic cell death (ICD) by chemotherapy unveils both danger signals (activating DCs) and non-mutated neoantigens (NM-neoAgs) in apoptotic tumor cells that are efficiently phagocytosed by DCs. NM-neoAgs derive by caspase cleavage of a variety of tumor antigens, are efficiently processed and (cross-)presented by DCs in form of peptides on MHC molecules (67) to T cells. CD8^+^ T cells proliferate and further expand in response to ICB treatment (anti-PD-1 therapy), providing improvement of effector function and conversion of PD-1^+^ T cells into PD-1^–^ T cells. Effector CD8^+^ T_EM_ cells can migrate into the tumor microenvironment and be further boosted by NM-neoAgs derived from chemotherapy-induced apoptotic tumor cells cross-presented by tissue DCs: CD8^+^ T_EM/EMRA_ cells provide tumor control through the by-stander effect of strong inflammatory cytokines and the recruitment of other T cells and innate immune cells (macrophages, neutrophils, natural killer cells,…). This scenario is supported by the evidence showing that NM-neoAg-specific CD8^+^ T cells are significantly represented in NSCLC patients previously submitted to chemotherapy, increase in their frequency upon ICB therapy, and correlated with overall survival (67). The addition of therapeutic NM-neoAg-based vaccines may improve T cell memory and tumor immunity, as well as convert cold into hot tumors.

The triple combination of immunogenic cell death by chemotherapy, vaccination with the resulting non-mutated neoantigens (i.e., generated following chemotherapy-induced apoptosis of tumor cells), and ICB treatment switching off the inhibitory T cell signals, may result in being beneficial in the immunotherapy of “cold” tumors, such as small cell lung cancer, MSS-colorectal cancer or MSS-hepatocellular carcinoma, characterized by DNA stability, effective mismatching repair mechanisms, low generation of mutated neoantigens, and very low number of TILs, in order to convert them into hot tumors (**Figure 3**). This combination may also provide beneficial effects in those hot tumors, in which TILs specific to mutated neoantigens became fully exhausted, in order to generate new tumor-specific immune responses and memory. Definitely, this combinatorial module may represent a tremendous resource for a new tumor immunotherapy approach providing the essential signals (1, 2, and 3) required for optimal T cell memory development (**Figure 3**).

Finally, systems to dampen Treg-mediated suppression of immune responses have been considered as further therapeutic approaches to verify their effect in improving immunological memory, for instance through targeting CD25, CTLA-4, or CCR4 on Tregs in order to deplete intratumoral Tregs (90–92). More recently, data on the metabolic profile of activated Tregs proposed that metabolic drugs targeting specific molecules of lipid turnover may preferentially modulate Tregs compared to other T cells (80).

## T Cell Memory and Tregs in Autoimmunity

A further important question is: how do most autoimmune diseases persist for several years in patients, despite the fact that immune responses are not conditioned by persisting (non-self) “invaders”, but presumably by a breakdown of peripheral immunological tolerance (e.g., anergy, exhaustion, Treg suppression) causing the unleash and activation of diverse autoaggressive B and T cells against self-antigens? Might the same immunoregulatory mechanisms ultimately maintaining the long-lasting relationship between persisting “invaders” (chronic infections or tumors) and the host, fine-tune the autoimmune responses, thus allowing that the final failure/destruction of the self-organs or tissues by the autoaggressive responses is delayed for many years?

Chronic TCR signaling is common in chronic infection, cancer, and autoimmunity, but the persisting antigens providing TCR signaling are generally different in the three contexts: (non-self) infectious antigens in chronic infection, (non-self) neoantigens in cancer, and self-antigens in autoimmunity. A main paradigm at the center of immunology is that, in the periphery, non-self-antigens (infectious or neoantigens) are recognized by high affinity TCRs (which are positively selected in the thymus and migrate in the periphery), whereas the self-antigens by low-affinity TCRs (high affinity T cells for the “self” having been purged in the thymus). An alternative, but not mutually exclusive route leading to autoimmunity is based on the evidence that not necessarily the antigenic targets of the so-called autoimmune diseases are self-antigens, but they can also be represented by neo(ex-self)-antigens generally generated by post-translational modifications of self-antigens in the periphery. Prototypical examples are neo(ex-self)-antigens in type 1 diabetes (T1D) (i.e., tissue transglutaminase-dependent deamidation or alternative-reading-frame-encoding of pro-insulin peptides), or in rheumatoid arthritis (i.e., citrullination or deamination of vimentin, lamin B1, non-muscle myosin, actin and other cytoskeleton or nuclear self-antigens) (93–98). In this perspective, these neo(ex-self)-antigens are recognized by non-self-specific T cells expressing high affinity TCRs, which are positively selected in the thymus and migrate in the periphery, because neo(ex-self)-antigens would not be expressed or expressed at concentrations that are not enough to delete them in the thymus. As a consequence, chronic stimulation with non-self-antigens (infectious or tumoral) or neo(ex-self)-antigens (modified self-antigens) should cause T cell exhaustion more efficiently than chronic stimulation with native self-antigens, with divergent impact in the development of the immunological memory. Consistent with this hypothesis, recent data in both experimental and human T1D showed that self- or non-self-reactive T cells shared common phenotypic, transcriptional and epigenetic program features of exhaustion, those autoreactive displayed a wider level of heterogenicity, depending on the TCR affinity for self-antigens (99, 100). Therefore, we could envisage that chronic tissue damage in autoimmunity may be established by alternate waves of self-reactive (the minority that have been able to escape central tolerance in the thymus) or neo(ex-self)-reactive T cells with moderate/high affinity TCRs, and self-reactive T cells with low affinity TCRs that are not purged in the thymus. In particular, recent data demonstrated that self-reactive or neo(ex-self)-reactive T cells with high affinity TCRs are more harmful and can evade peripheral Treg-mediated tolerance (e.g., by counter-suppressing Tregs) (98), but they should be more susceptible to exhaustion than those with low affinity TCRs, due to the stronger stimulation by persistent self- or neo(ex-self)-antigens. By contrast, self-reactive T cells with low affinity TCRs are significantly less harmful than the former, and are efficiently controlled by Tregs, contributing hence to maintain a state of chronic low-level inflammation (98). Thus, the persistence of self-antigens or neo(ex-self)-antigens conditions, in the long run, promote exhaustion of specific T cells with high affinity TCRs, and Treg-mediated suppression of those with low affinity TCRs. The alternate fluctuation by self-reactive or neo(ex-self)-antigens T cells with high and low affinity TCRs may in part explain the clinical outcome of autoimmune diseases characterized by a chronic alternation of acute and quiescent phases that finally undergo tissue destruction after a long time. This scenario could account for results showing that, unlike in chronic infections or tumors, CD8^+^ T cell exhaustion is associated with a good outcome and a low risk of relapse in autoimmune diseases, proposing that manipulation of exhaustion may represent a novel therapeutic strategy to suppress autoreactivity by using agonists of ICs (e.g., CTLA-4, PD-1, TIM-3, LAG-3), or antagonists of activating receptors (e.g., CD28, OX40, GITR, CD137) (99, 100).

Regarding the role of Tregs, if the aim is to inhibit them in chronic infections or tumors, *vice versa* the therapeutic goal is the induction or activation of Tregs in autoimmune diseases by various approaches, namely, the transfer of autologous *in vitro*-expanded Tregs to suppress autoimmune responses (101), or induction of Tregs directly *in vivo* by administration of immunocomplexes of IL-2 and specific anti-IL2 antibody selectively promoting the expansion of Tregs (expressing the high-affinity trimeric IL-2R that includes IL-2Rα) without expanding activated effector T cells (102, 103).

## Concluding Remarks

Immunological memory is a major and unique resource of the adaptive immunity allowing to remember for a long time the antigens that the individuals encounter, and to promptly respond on demand in the case of antigen re-encounter. The molecular mechanisms governing immunological memory in the different B or T cell subsets at the transcriptional and epigenetics level, are revealing fundamental pathways. The generation of new selective compounds capable to influence the immunological memory (improvement or suppression) may become extremely useful in the therapy of the different pathological contexts in the next years. The major goal in chronic infection or cancer is to restore protective immune responses that have been made dysfunctional by excessive exhaustion and Treg-suppression, in order to help eliminate persistent pathogens or tumors providing chronic stimulation, and to develop long-term immunological memory controlling possible re-emergences of the primary infections or tumors. By contrast, the major goal in autoimmunity is exactly the opposite, namely to restore the tolerance of the persisting self-antigens through the use of agonists of T cell ICs, antagonists of T cell activating receptors, or by reinvigorating Tregs, so as to convert the picture of autoimmune aggression into that of exhaustion and suppression of autoreactive B or T cells, and to keep the autoimmunological memory under tone.

## Author Contribution

VB ideated and wrote this review.

## Funding

This work was supported by the following grants: Associazione Italiana per la Ricerca sul Cancro (AIRC) (progetti “Investigator Grant” [IG]-2014 id. 15199 and IG-2017 id. 19939 to VB); The Accelerator Award 2018 (Project 620 Id. 22794 to VB); Fondazione Italiana Sclerosi Multipla (FISM) onlus (cod. 2015/R/04 and 2019/R-Single/053 to VB); Ministero della Salute (Ricerca finalizzata [RF-2010-2310438 and RF 2010-2318269] to VB); Ministero dell’Istruzione, dell’Università e della Ricerca (MIUR) (PRIN 2010–2011 prot. 2010LC747T_004 to VB); Fondo per gli investimenti di ricerca di base (FIRB)-2011/13 (no. RBAP10TPXK to VB); Istituto Pasteur Italia—Fondazione Cenci Bolognetti (grant 2014–2016); International Network Institut Pasteur, Paris—”Programmes Transversaux De Recherche” (PTR n. 20-16).

## Conflict of Interest

The author declares that the research was conducted in the absence of any commercial or financial relationships that could be construed as a potential conflict of interest.

## Publisher’s Note

All claims expressed in this article are solely those of the authors and do not necessarily represent those of their affiliated organizations, or those of the publisher, the editors and the reviewers. Any product that may be evaluated in this article, or claim that may be made by its manufacturer, is not guaranteed or endorsed by the publisher.

## Abbreviations

APCs, antigen-presenting cells; DC, dendritic cell; MHC, major histocompatibility complex; TCRs, T cell receptors; L, ligand; h, helper; c, cytotoxic; PAMP, pathogen-associated molecular pattern; DAMP, danger-associated molecular pattern; T_N_, naïve T; T_CM_, central memory T; T_EM_, effector memory T; T_PM_, peripheral memory T; T_RM_, resident memory T; HAV, hepatitis A virus; TFs, transcription factors; ICs, immune check-points; Tregs, regulatory T cells; NSCLC, non-small cell lung cancer; TILs, tumor-infiltrating T cells; ICB, IC blockade; T1D, type 1 diabetes.
